# *Candida* Extracellular Nucleotide Metabolism Promotes Neutrophils Extracellular Traps Escape

**DOI:** 10.3389/fcimb.2021.678568

**Published:** 2021-07-13

**Authors:** Mariana Afonso, Ana Rita Mestre, Guilherme Silva, Ana Catarina Almeida, Rodrigo A. Cunha, José Roberto Meyer-Fernandes, Teresa Gonçalves, Lisa Rodrigues

**Affiliations:** ^1^ Medical Microbiology Research Group, CNC-Center for Neurosciences and Cell Biology, Coimbra, Portugal; ^2^ FMUC-Faculty of Medicine, University of Coimbra, Coimbra, Portugal; ^3^ Purines at CNC - Neuromodulation Group, CNC-Center for Neurosciences and Cell Biology, Coimbra, Portugal; ^4^ Institute of Medical Biochemistry Leopoldo de Meis, Federal University of Rio de Janeiro, Rio de Janeiro, Brazil

**Keywords:** *Candida*, neutrophils, nucleotide metabolism, nucleotidase, neutrophil extracellular traps escape

## Abstract

Host innate immunity is fundamental to the resistance against *Candida albicans* and *Candida glabrata* infection, two of the most important agents contributing to human fungal infections. Phagocytic cells, such as neutrophils, constitute the first line of host defense mechanisms, and the release of neutrophil extracellular traps (NETs) represent an important strategy to immobilize and to kill invading microorganisms, arresting the establishment of infection. The purinergic system operates an important role in the homeostasis of immunity and inflammation, and ectophosphatase and ectonucleotidase activities are recognized as essential for survival strategies and infectious potential of several pathogens. The expression and unique activity of a 3′-nucleotidase/nuclease (3′NT/NU), able to hydrolyze not only AMP but also nucleic acids, has been considered as part of a possible mechanism of microbes to escape from NETs. The aim of the present study was to evaluate if yeasts escape from the NET-mediated killing through their 3′NT/NU enzymatic activity contributing to NET-hydrolysis. After demonstrating the presence of 3′NT/NU activity in *C. albicans*, *C. glabrata*, and *Saccharomyces cerevisiae*, we show that, during neutrophils-*Candida* interaction, when NETs formation and release are triggered, NETs digestion occurs and this process of NETs disruption promoted by yeast cells was prevented by ammonium tetrathiomolybdate (TTM), a 3′NT/NU inhibitor. In conclusion, although the exact nature and specificity of yeasts ectonucleotidases are not completely unraveled, we highlight the importance of these enzymes in the context of infection, helping yeasts to overcome host defenses, whereby *C. albicans* and *C. glabrata* can escape NET-mediate killing through their 3′NT/NU activity.

## Introduction

Fungi are ubiquitous microorganisms able to interact with humans in multiple ways (Baxi et al., 2016; Firacative, 2020). The interactions between host cells and microbes will define whether the microbe can evolve into pathogenicity, or colonize passively the human host or will be eradicated by the immune system (Hube, 2009). The dynamics of this interaction may vary among different fungal species and morphologies and may also be intrinsically influenced by the host’s defense mechanisms and the associated immune responses (Romani, 2011; Sparber and Leibundgut-Landmann, 2017). Two of the most pathogenic fungi belonging to the genus *Candida*, *Candida albicans* and *Candida glabrata*, are considered important opportunistic agents of fungal infections accounting for approximately 90% of invasive infections in North America (Negoro et al, 2020), in view of their variability and adaptability (Pfaller and Diekema, 2007; Brunke and Hube, 2013).

Innate immunity is fundamental in the resistance against pathogenic microorganisms, with phagocytic cells playing important roles during host infection, killing or damaging the fungal pathogens (Shoham and Levitz, 2005; Erwig and Gow, 2016). Neutrophils, human phagocytes of the first line of defense, are crucial in antifungal mechanisms, generating several responses when meeting microbes, such as degranulation and phagocytosis (Kumar and Sharma, 2010; Amulic et al., 2012; Bardoel et al., 2014). Neutrophil-mediated killing also occurs upon the release of neutrophil extracellular traps (NETs), which are web-like structures, composed by granular and nuclear constituents (like DNA and histones). Two types of NETosis are described, lytic and non-lytic (Yang et al., 2016; Papayannopoulos, 2018), representing an important strategy to immobilize and kill invading microorganisms, preventing the establishment of infection (Brinkmann et al., 2004; Kaplan and Radic, 2012; Branzk et al., 2014; Dainichi et al., 2020).

The purinergic signaling system involves the release of ATP and adenosine to the extracellular milieu during cellular disturbances, both important controllers of the immune cell functions (Burnstock and Verkhratsky, 2009; Antonioli et al., 2013; Virgilio and Vuerich, 2015; Cekic and Linden, 2016). In neutrophils, ATP induces neutrophil activation, but the accumulation of adenosine through extracellular ATP degradation can lead to considerable inhibitory and anti-inflammatory effects (Antonioli et al., 2013; Xu et al., 2019). In addition, several studies reveal that this sequential dephosphorylation of ATP into adenosine (ATP > ADP > AMP > adenosine) is under control of ectonucleotidases, cell surface-located enzymes. These ecto-enzymes are essential for pathogen survival strategies and virulence factors, such as host-parasite interaction and yeast adhesion to epithelial cells, being determinant in the microorganism infection (Borges et al., 2007; Marques-da-Silva et al., 2008; Paletta-Silva et al., 2011; Russo-Abrahão et al., 2011; Freitas-Mesquita and Meyer-Fernandes, 2017). Most studies have concentrated on one ectonucleotidase, ecto-5′nucleotidase (CD73), which was shown to have a robust immunomodulatory activity (Eberhardt et al., 2020; Harvey et al., 2020; Yu et al., 2020). Surprisingly, we previously observed that *C. albicans* do not present a classical ecto-5′nucleotidase profile and that these yeasts are also able to use 3′AMP as substrate, although without a specific or effective assigned role (Rodrigues et al., 2015). Notably, the expression and specific action of such a 3′-nucleotidase/nuclease (3′NT/NU) has been described as being part of a possible mechanism of microorganisms to escape NETs (Guimarães-Costa et al., 2014; Freitas-Mesquita et al., 2019). In *Leishmania*, the study of 3′NT/NU showed that this enzymatic activity allowed the parasite to escape from the toxic effects of NETs (Guimarães-Costa et al., 2014). Therefore, we now raised the hypothesis that a putative 3′NT activity could also be a mechanism used by *C. albicans* and other yeasts to escape NETs. Thus, the aim of the present work was to directly test if 3′NT/NU enzymatic activity is involved in the ability of these yeast species to escape neutrophils control, in particular how its 3′NT/NU enzymatic activity can impact in NETs and NETosis escape.

## Materials and Methods

### Strains, Media, and Growth Conditions

*Candida albicans* YP0037, *Candida glabrata* YP0937, and *Saccharomyces cerevisiae* YP0467 strains were obtained from the Microbiology Pathogenic Yeast Collection, University of Coimbra. Yeasts were grown overnight at 30°C on YPD (0.5% yeast extract, 1% peptone, 2% agar, and 2% glucose) agar plates, harvested by centrifugation, and resuspended in phosphate-buffered saline (PBS) (pH 7.4). Whenever necessary, yeasts were pre-labeled with 1 μM Oregon Green 488 (Invitrogen), for 30 min at 30°C with continuous gentle shaking in the dark, and then washed twice with PBS containing 100 mM glycine, or heat-killed (HK) at 95°C, for 30 min. Yeast cells were counted in a Neubauer chamber and adjusted to the desired cell concentration.

### Ectonucleotidase Activity

Ectonucleotidase activity was determined by the rate of inorganic phosphate (Pi) released, as previously described (Rodrigues et al., 2015). Briefly, intact cells (3 x 10^9^ cells) were incubated for 1 h at room temperature (RT) in 0.5 ml of reaction mixture containing 116 mM NaCl, 5.4 mM glucose, 50 mM HEPES-MES-Tris buffer (pH 4.0), and 5 mM of 5′AMP (Sigma-Aldrich) or 5 mM of 3′AMP (Sigma-Aldrich) as substrates. Additional tubes with substrates incubated with the ectonucleotidase inhibitor ammonium tetrathiomolybdate (TTM; 100 µM) were also prepared. The reaction was stopped by the addition of 1 ml of 25% charcoal in 0.1 M HCl. Then, the mixture was centrifuged and 0.1 ml of the supernatant was added to 0.1 ml of Fiske Subbarow reactive mixture. The absorbance of the released Pi was measured spectrophotometrically at 650 nm. Ecto-3′ and ecto-5′-nucleotidase activities were calculated by subtracting the nonspecific 3′AMP and 5′AMP hydrolysis in blanks; the concentration of Pi released in the reaction was determined using a standard curve of Pi.

### Isolation of Human Neutrophils

Human polymorphonuclear cells (PMNs) were obtained from whole-blood samples of healthy young volunteer donors and collected in a lithium heparin vacutainer. Using a well-established methodology, blood samples were carefully transferred to falcon tubes and diluted in PBS solution at 1:1 ratio, followed by dextran sedimentation (3% dextran solution in PBS) of red blood cells (RBC), during 90 min at 37°C in a 5% CO_2_ atmosphere. The PMN-rich supernatant was carefully aspirated into a new tube, diluted in PBS solution and centrifuged 5 min at 878*g*, 4°C. The supernatant was discarded and the remaining pelleted RBC were lysed with RBC lysis buffer (150 mM NH_4_Cl, 10 mM KHCO_3_, 0.1 mM EDTA). The tube content was mixed by inversion, and lysis was then stopped with addition of PBS solution at 1:1 ratio. After centrifugation for 10 min, 319*g*, 4°C, the supernatant was carefully discarded and the white pellet with PMN cells was resuspended in RPMI 1640, 1% PenStrep antibiotic mix (Sigma-Aldrich), and counted in a Neubauer chamber.

### Infection Assays

Fresh human neutrophils (1 × 10^6^ cells/well) were seeded in a 12-well plate treated with poly-l-lysine (with or without 16 mm glass coverslips) and allowed to sediment for 1 h at 37°C in a 5% CO_2_ atmosphere. Sedimented neutrophils were then washed with PBS and incubated with *C. albicans*, *C. glabrata*, or *S. cerevisiae* (multiplicity of infection [MOI], 1:1), and with the NETs inducer phorbol 12‐myristate 13‐acetate (PMA; EMD Millipore), 100 nM, at 37°C, in a 5% CO_2_ atmosphere.

### Immunofluorescence and Microscopy Analysis

At the end of each infection period (1 or 3 h), coverslips were fixed with 4% paraformaldehyde (PFA) in PBS for 15 min at RT and washed. Cells were stained with Wheat Germ Agglutinin (WGA), tetramethylrhodamine conjugate (5 μg/ml, Invitrogen), washed, and neutrophils nuclei stained with 4′,6-diamidino-2-phenylindole (DAPI) for 5 min at RT. Coverslips were mounted on glass slides with DAKO mounting medium (DakoCytomation Fluorescent Mounting Medium). For each experiment, duplicates for each condition were performed, and five digital images were obtained per coverslip with a Zeiss Axio Observer Z1 fluorescence microscope, with Plan-ApoChromat 20× and 63×/1.40 immersion objectives. Zeiss Zen lite and Image J software were used to analyze the images.

### Neutrophils Viability Assays

To check the neutrophils viability/membrane integrity, a Trypan Blue exclusion test was performed. Briefly, the infection assays were performed as mentioned above and, after incubation, the cells were washed and scraped, and the obtained cell suspension mixed with a 4% Trypan Blue solution. Viable and non-viable cells were counted in a Neubauer chamber.

### Yeasts Viability Assays

*C. albicans*, *C. glabrata*, and *S. cerevisiae* viability was evaluated using a colony forming unit (CFU) assay. Neutrophils (2.5 × 10^5^ cells/well) were seeded in 96-well plates coated with poly-l-lysine and incubated with yeasts for 1 h at 37°C in a 5% CO_2_ atmosphere. The supernatants were collected, corresponding to the non-internalized yeasts (“out” condition). The adhered neutrophils were then lysed with 0.5% Triton X-100 in sterile distilled water and scrapped. The yeasts recovered in this fraction corresponded to the adhered and internalized yeasts (“in” condition). Serial dilutions were performed and each condition was spread in YPD agar plates, with colonies counting after 3 days at 30°C.

### NETs Digestion Assays

Human neutrophils prepared as described above were incubated for 3 h at 37°C in a 5% CO_2_ atmosphere with the NETs inducer PMA (100 nM). NET-enriched supernatants (pH 7-8) were collected and incubated with *C. albicans*, *C. glabrata*, or *S. cerevisiae* to evaluate the ability of yeasts to degrade NETs through their 3′NT/NU enzymatic activity. Yeast cells (1 × 10^6^ cells/well), treated or not with the 3′NT/NU inhibitor TTM (100 μM), were incubated with the NET-enriched supernatants for 3 h at 37°C. After this period, some samples were also treated with EcoRI and HindIII restriction enzymes (20 U/ml) for 30 min. The supernatants of each condition were collected and centrifuged at 1,520*g* for 10 min at 4°C, concentrated in a freeze-drying apparatus and run in a GreenSafe pre-stained 1% agarose gel.

### Statistical Analysis

Data analysis was performed using GraphPad Prism software (Version 7.04). Data are presented as means ± SEM. Statistical differences were determined, depending on the analysis, using one- or two-way ANOVA with Bonferroni post-hoc test or unpaired t test for analysis of two groups. Results with P < 0.05 were considered statistically significant.

## Results

**Figure 1:**
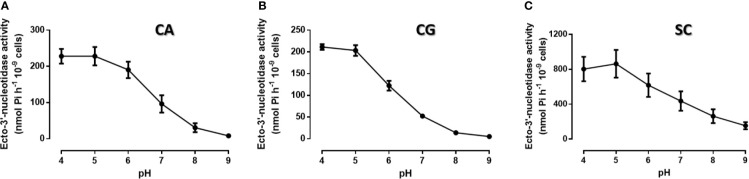
Ecto-3′-nucleotidase activity at different pH conditions. Intact cells of *C. albicans* (**A**, CA), *C. glabrata* (**B**, CG) and *S*. *cerevisiae* (**C**, SC) were allowed to cleave 3′AMP for 1 h at room temperature, at different pH conditions. All yeasts present maximum ecto-3′-nucleotidase activity at pH 4.0. Data are shown as means ± SEM of at least three independent experiment.

**Figure 2:**
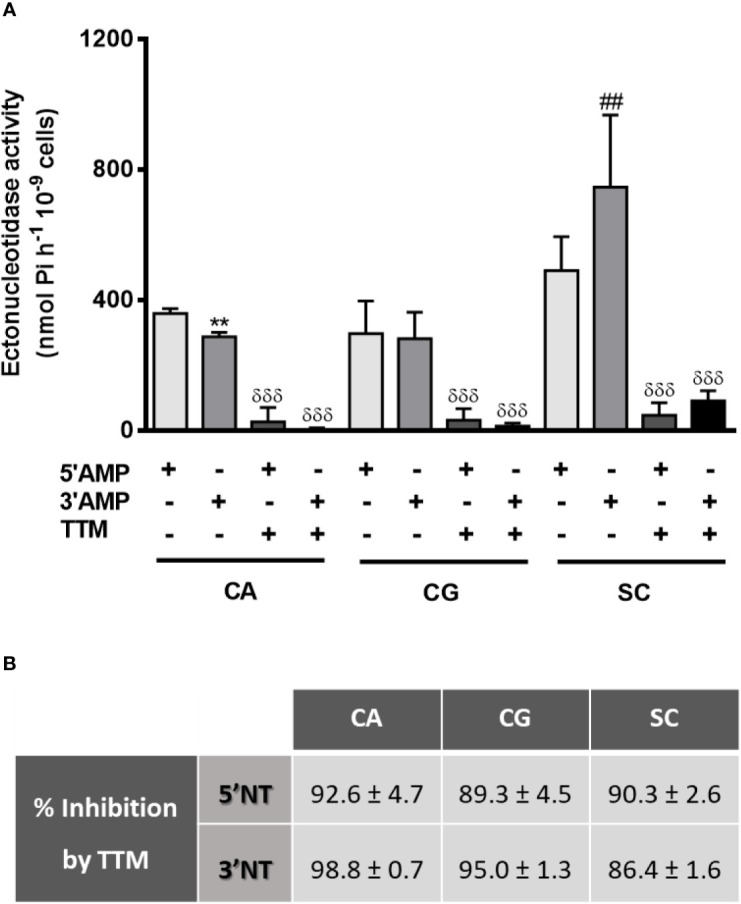
Yeasts ectonucleotidases activity with TTM treatment. Intact cells of *C. albicans* (CA), *C. glabrata* (CG) and *S*. *cerevisiae* (SC) were incubated for 1 h at room temperature with 5′AMP, 3′AMP or TTM inhibitor together with each substrate **(A)**. Percentage of TTM inhibition for each condition is presented in **(B)**. Data are shown as means ± SEM of at least three independent experiments. **p<0.01, CA 3′AMP *vs.* CA 5′AMP; ^##^p<0.01, SC 3′AMP *vs.* SC 5′AMP; ^δδδ^p<0.001, TTM-treated yeasts *vs.* untreated.

We first determine yeasts ecto-3′-nucleotidase (3′NT) and ecto-5′-nucleotidase (5′NT) activities in *C. albicans*, *C. glabrata*, and *S. cerevisiae* (**Figures 1**, **2**). Yeast 3′NT activity was higher under acidic pH levels (higher activity at pH 4) and decreased as pH increased to higher alkalinity, in the three yeast species studied (**Figure 1**). Further studies were performed under the optimal pH 4 and, after 1 h incubation period, intact *C. albicans, C. glabrata*, and *S. cerevisiae* cells were able to hydrolyse both 5′AMP and 3′AMP substrates (**Figure 2A**). When yeasts were incubated with ammonium tetrathiomolybdate (TTM), a 3′nucleotidase/nuclease (3′NT/NU) inhibitor together with each substrate, a significant decrease in ectonucleotidase activities was observed (**Figure 2A**). For all yeasts, the incubation with TTM resulted in a consistent 5′NT and 3′NT/NU inhibition; for *C. albicans* and *C. glabrata*, the 3′NT/NU inhibition is even more marked (**Figure 2B**; p<0.001). The TTM concentration used, 100 μM, was selected based on inhibitory concentration curves obtained with *C. albicans*, the most pathogenic yeast used in this study (**Supplementary Figure 1**).

**Figure 3:**
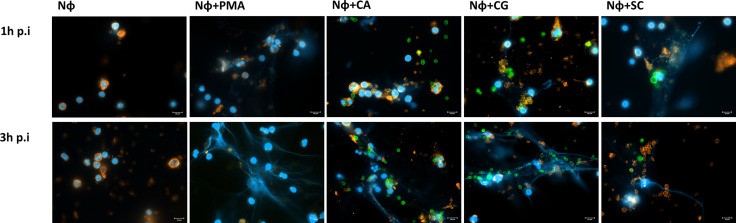
NETs stimulation upon interaction of isolated neutrophils with yeast cells. Neutrophils (Nɸ), neutrophils stimulated with PMA (Nɸ+PMA), neutrophils with *C. albicans* (Nɸ+CA), neutrophils with *C. glabrata* (Nɸ +CG), and neutrophils with *S. cerevisiae* (Nɸ+SC) were observed at 1 and 3 h post infection. The neutrophils’ cell membrane sialic acids were stained with WGA, tetramethylrhodamine conjugate (red), nucleic acids stained with DAPI (blue), and each yeast cell stained with Oregon Green (green). Images are representative of sets of different experiments and were captured at 63× magnification. Scale bars represents 10 μm.

Because the scarcely studied 3′NT/NU activity present in *C. albicans*, *C. glabrata*, and *S. cerevisiae* cells has been associated to the escape of other microorganisms from NETs control (Guimarães-Costa et al., 2014), we next tested how these yeasts interact with neutrophils and stimulate NETs formation. Using a fluorescence microscopy approach, we were able to observe the interactions established between neutrophils and yeasts, at 1 and 3 h post infection (p.i.) (**Figure 3**; representative images at 63× magnification). As a control, assays with only neutrophils (Nɸ) and neutrophils stimulated with phorbol myristate acetate (PMA) (Nɸ+PMA) were also performed. As expected, this stimulus clearly induced neutrophils to release NETs into the extracellular milieu, as testified by the increase of nucleic acids blue fluorescence smears/blurs corresponding to the NETs. Uninfected neutrophils (Nɸ) did not release NETs, although, in some cases, and most probably due to cell activation upon manipulation, a very slight increment in nucleic acids/NETs staining was occasionally observed. In what regards the infection of neutrophils with yeasts, *C. albicans* (Nɸ+CA), *C. glabrata* (Nɸ+CG), and *S. cerevisiae* (Nɸ+SC) were able to activate neutrophils and trigger NETs release, with this process being more evident at 3 h p.i. Surprisingly, during Nɸ+CA interaction, the activation of neutrophils and NETs release could be observed, but the NETs (in blue) poorly colocalize with *C. albicans* cells (in green); free/not trapped by NETs yeast were found more frequently, contrary to what was observed with *C. glabrata* and *S. cerevisiae* (**Figure 3**).

**Figure 4:**
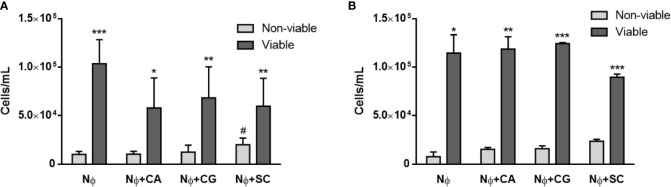
Neutrophils’ viability upon yeasts interaction. Trypan blue assays were performed to distinguish between viable and non-viable neutrophils (Nɸ) at 1 h **(A)** and 3 h **(B)** post infection with *C. albicans* (CA), *C. glabrata* (CG) and *S*. *cerevisiae* (SC). Data represent at least three independent experiments (means ± SEM). *p<0.05, **p<0.01, ***p<0.001, in viable *vs.* non-viable conditions; ^#^p<0.05, *vs.* non-viable Nɸ.

**Figure 5:**
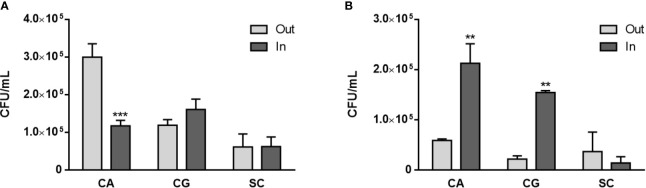
Yeasts viability upon neutrophils interaction. *C. albicans* (CA), *C. glabrata* (CG) and *S*. *cerevisiae* (SC) colony forming unit (CFU) assays were performed at 1 h **(A)** and 3 h **(B)** post infection: supernatants were collected (“out” columns) to access non-adhered/phagocytosed yeasts, and adhered neutrophils were lysed to release adhered/phagocyted yeasts (“in” columns). Colonies were counted after 3 days, and data represent at least three independent experiments (means ± SEM). **p<0.01, ***p<0.001, in “in” *vs.* “out” conditions.

Once yeasts have stimulated neutrophils to release NETs, the viability of both cell types is expected to be affected, and this was quantified next. A Trypan blue exclusion test revealed that the number of viable neutrophils at 1 and 3 h p.i. was always statistically higher than the number of non-viable neutrophils for all the conditions tested (**Figure 4**). Nonetheless, with *S. cerevisiae* an increase in the number of non-viable cells was found at 1 h p.i. (**Figure 4A**), when compared with control neutrophils (p<0.05); although not statistically significant, a decrease in the number of viable cells at 3 h p.i. also seems to occur (**Figure 4B**). Colony-forming unit (CFU) assays were performed to assess yeast viability (**Figure 5**). At 1 h p.i. (**Figure 5A**), *C. albicans* (CA) presented the highest viability values for yeasts not adhered or not phagocytized by neutrophils and/or retained in NETs (“out” columns), when compared with the other yeast species, and a statistical inferior number in its own adhered/phagocytized/retained viable cells (“in” *vs.* “out” columns; p<0.001), thus corresponding to a higher ability to escape neutrophils’ immune responses. *C. glabrata* (CG) and *S. cerevisiae* (SC) presented lower CFU counts, being more effectively controlled by neutrophils, with no statistical differences between adhered *versus* non-adhered viable cells. In more prolonged interactions, at 3 h p.i. (**Figure 5B**), both *C. albicans* (CA) and *C. glabrata* (CG) presented higher viability counts of adhered/phagocytized/retained cells when compared with non-adhered ones (“in” *vs*. “out” column; p<0.01), and those values were again more evident in *C. albicans* (CA). Being the less virulent species used, *S. cerevisiae* (SC) presented lower viability counts in all conditions tested, being more prone to neutrophil elimination.

**Figure 6:**
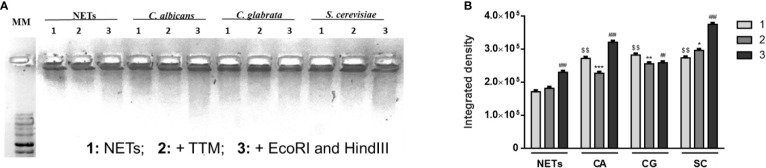
NETs digestion in the presence of yeasts. NET-enriched supernatants were used under different conditions: NETs, NETs with *C. albicans* (CA), NETs with *C. glabrata* (CG), and NETs with *S. cerevisiae* (SC) (1); each condition was also co-incubated with 100 μM TTM (2) for 3 h and restriction enzymes (EcoRI and HindIII) were added at each condition for an additional 30 min period (3). Samples were run on a 1% GreenSafe agarose gel to evaluate yeast’s ability to digest nucleic acids and NETs (**A**; representative image of a set of different experiments) and nucleic acids smears integrated density (mean gray values x area) were quantified **(B)**. $$p<0.01, yeasts-NETs *vs.* neutrophil-NETs; *p<0.05, SC TTM treatment (2) *vs.* SC NETs (1); **p<0.01, CG TTM treatment (2) *vs.* CG NETs (1); ***p<0.001, CA TTM treatment (2) *vs.* CA NETs (1); ^##^p<0.01, CG restriction enzymes treatment (3) *vs.* CG NETs (1); ^###^p<0.001, NETs, CA, and SC restriction enzymes treatment (3) *vs.* NETs, CA, and SC NETs (1), respectively.

**Figure 7:**
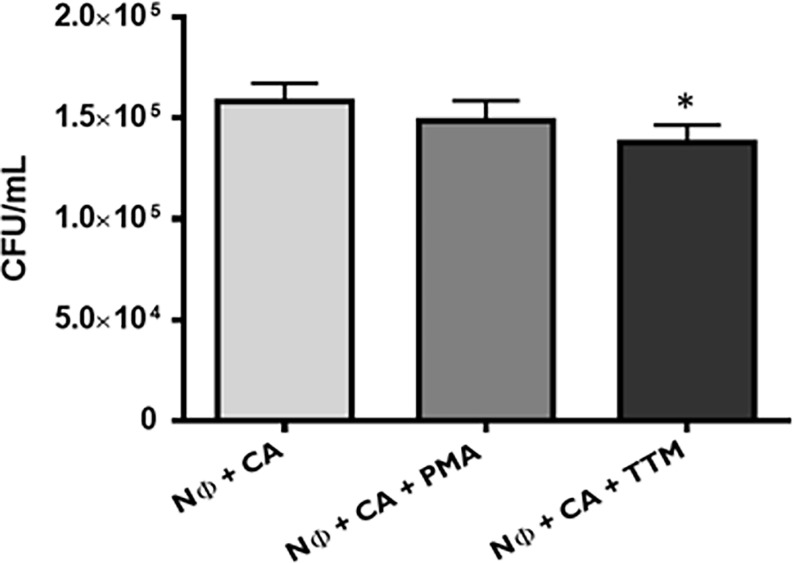
*Candida albicans* viability upon neutrophils interaction. *C. albicans* (CA) colony forming unit (CFU) assays were performed at 1 h post infection, upon different conditions: neutrophils with CA (Nɸ+CA) and neutrophils with CA, stimulated with PMA (Nɸ+CA+PMA) or TTM (Nɸ+CA+TTM). Colonies were counted after 3 days, and the data represent at least three independent experiments (means ± SEM). *p<0.05.

**Figure 8:**
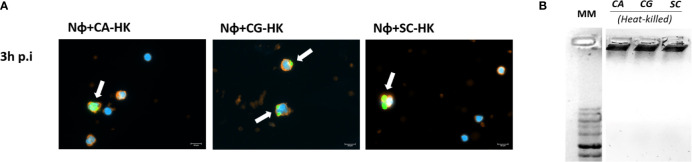
Interaction of neutrophils with heat-killed (HK) yeast cells. **(A)** Neutrophils with HK-*C. albicans* (Nɸ+HK-CA), neutrophils with HK-*C. glabrata* (Nɸ+HK-CG), and neutrophils with HK-*S. cerevisiae* (Nɸ+HK-SC) were observed at 3 h post infection. Neutrophils’ cell membrane sialic acids were stained with WGA, tetramethylrhodamine conjugate (red), nucleic acids stained with DAPI (blue), and each yeast cell was stained with Oregon Green (green). White arrows indicate clusters of phagocytized yeasts. Images are representative of sets of different experiments and were captured at 63× magnification. Scale bars represents 10 μm. **(B)** CA-HK, CG-HK, and SC-HK were incubated with NETs-enriched supernatants during 3 h. Samples were run on a 1% GreenSafe agarose gel to evaluate yeasts ability to digest nucleic acids and NETs (representative image of a set of different experiments).

These differences in terms of viability were further studied regarding the ability of yeasts to digest NETs, through the 3′NT/NU enzymatic activity, in an attempt to unravel this new route of escape against neutrophils response mechanisms. For this, NET-enriched supernatants were obtained (with measured pH between 7 and 8) and used to assess the ability of *C. albicans* (CA), *C. glabrata* (CG), and *S. cerevisiae* (SC) to digest nucleic acids and, consequently NETs (**Figure 6**). Based on the premise that this digestion would led to the cleavage of nucleic acids from NETs into smaller fragments, the product of these digestion reactions was run in electrophoresis gels, checking for differences in the smear profile (**Figure 6A**). This confirmation was done by quantification of the corresponding integrated densities of each lane (taking into consideration mean gray values × area; **Figure 6B**). A control with co-incubation with EcoRI and HindIII restriction enzymes was performed in all conditions, showing a clear DNA NETs cleavage corresponding to higher gel smears and higher DNA quantifications in each condition. The lanes with NETs plus *C. albicans* (CA), *C. glabrata* (CG), and *S. cerevisiae* (SC) displayed more pronounced DNA smears and correspondent integrated densities when compared with neutrophils not exposed to yeasts (p<0.01), thus proving the ability of yeasts to degrade NETs. However, when yeast cells were incubated with the 3′NT/NU inhibitor, TTM, which has no impact in neutrophils viability (**Supplementary Figure 2**), a decreased ability to digest NETs was observed in *C. albicans* (CA; p<0.001) and *C. glabrata* (CG; p<0.01), as demonstrated by smaller gel smears and lower integrated density quantification. This profile was less evident in *S. cerevisiae* (SC; p<0.05). Moreover, *C. albicans*, the most virulent yeast species, showed a decreased yeast viability when the cells were incubated with TTM (**Figure 7**). The incubation of NETs with heat-killed (HK) *C. albicans*, *C. glabrata*, and *S. cerevisiae* cells did not result in nucleic acid degradation, as inferred from the lack of DNA smear (**Figure 8A**). Furthermore, HK yeasts induced neutrophils’ phagocytosis and residual NETs formation instead of a visible NETs induction as seen with live yeasts (**Figure 8B**).

## Discussion

At the time of an infection, innate immunity is crucial in the host resistance against fungal pathogens, killing or damaging the invading microorganisms (Shoham and Levitz, 2005; Erwig and Gow, 2016). During an infection, *C. albicans* and *C. glabrata* both induce proinflammatory immune responses, which involve the recruitment of phagocytic cells, especially neutrophils. These cells are valuable for their clearance, early in the course of infection (Pappas et al., 2018), as essential effector cells in the control and elimination of invading pathogens (Kumar and Sharma, 2010; Amulic et al., 2012; Gazendam et al., 2016). At the site of infection, neutrophils possess a set of carefully regulated mechanisms, such as phagocytic uptake (Amulic et al., 2012; Bardoel et al., 2014; Gazendam et al., 2016; Castanheira and Kubes, 2019) or release of neutrophil extracellular traps (NETs) (Brinkmann and Zychlinsky, 2012; Yang et al., 2016; Papayannopoulos, 2018). A role for NETs in the response to *Candida* species was already described; NETs were induced when in contact with yeast and hyphal forms both in *C. albicans* (Urban et al., 2006; Urban et al., 2009; Zawrotniak et al., 2017; Wu et al., 2019; Zawrotniak et al., 2019) and in *C. glabrata* (Johnson et al., 2017), revealing an influence in the control of candidiasis (Urban and Nett, 2019). Nevertheless, a diversity of fungi has the capacity to develop resistance and escape NETs control (Urban and Nett, 2019). One mechanism associated with resistance may be related to the sequential dephosphorylation of ATP into adenosine by ectophosphatases and ectonucleotidases, which relevance is increasingly recognized in the modulation of the immune response (Antonioli et al., 2013) and in pathogen virulence factors (Borges et al., 2007; Marques-da-Silva et al., 2008; Freitas-Mesquita and Meyer-Fernandes, 2017; Paletta-Silva et al., 2011; Russo-Abrahão et al., 2011). This purinergic system is also essential for pathogen survival strategies, as demonstrated in the association of 3′-nucleotidase/nuclease (3′NT/NU) with the mechanism of microorganisms that escape from NETs (Guimarães-Costa et al., 2014; Freitas-Mesquita et al., 2019). The initial findings we previously described (Rodrigues et al, 2015) led us to pursue deeper studies on 5′NT and 3′NT/NU enzymatic activities to further understand a putative yeast escape to NETosis through 3′NT/NU in distinct pathogenic yeasts, *C. albicans*, *C. glabrata*, compared with *Saccharomyces cerevisiae*, as a less pathogenic model organism (Belda et al., 2019).

We now show, for the first time, that *C. glabrata*, and *S. cerevisiae* possess the biological trait of being able to use 5′AMP and 3′AMP as extracellular substrates, as described before in *C. albicans* (Rodrigues et al., 2015). When incubated with a 3′NT/NU inhibitor, TTM, yeasts showed a decrease both in 3′AMP and 5′AMP hydrolysis: TTM is an analog of the inorganic salt ammonium molybdate, which has previously been also found to be a 5′NT inhibitor (Borges et al., 2007; Freitas-Mesquita and Meyer-Fernandes, 2017; Freitas-Mesquita et al., 2019). As expected, in the presence of TTM, the amount of extracellularly hydrolyzed 3′AMP was substantially decreased in all conditions, especially in *C. albicans*, with a 3′NT/NU inhibition of 98.8%. The analysis of data obtained with neutrophils infection with *C. albicans*, *C. glabrata*, and *S. cerevisiae* confirmed that all yeasts triggered NETosis, with the disintegration of the nuclear envelop and consequent release of decondensed chromatin into the cytoplasm, as compared with the control of neutrophils stimulated with PMA, a potent inducer of NETs. Several studies already demonstrated that *C. albicans* is a potential target of NETs and NETosis (Urban et al., 2006; Urban et al., 2009; Zawrotniak et al., 2017; Wu et al., 2019; Zawrotniak et al., 2019). In the results presented here, NETosis seem to be activated at early stages of infection, at an MOI of 1:1, in a possible rapid non-lytic NET formation (Castanheira and Kubes, 2019). This evidence is also consistent with the fact that, besides the size of the pathogen, the number of pathogenic cells possibly determines the defense mechanism engaged by the host; this is in accordance with the results obtained by others (Johnson et al., 2016; Zawrotnaik et al., 2017), describing that a large number of pathogenic cells can decrease NETosis. However, the exact type of NETosis pathway triggered by yeast cells is still not fully understood (Castanheira and Kubes, 2019), although vital NETosis was already reported in *C. albicans* (Byrd et al., 2013; Zawrotniak et al., 2017; Wu et al., 2019). Our results also showed that *C. glabrata* was able to trigger NETs release from 1 h p.i., in accordance with the work of Johnson et al. (2017); surprisingly, the less virulent *S. cerevisiae* was also able to trigger the release of NETs. Brankz et al. (2014) proposed that the larger cell size of *S. cerevisiae* may be a crucial factor for the release of NETs by neutrophils.

Regarding the neutrophils’ viability on interaction with yeast cells, no significant alterations were found among the conditions tested. The viability of neutrophils upon exposure to yeasts is an important indication on the type of NETosis, lytic or non-lytic (Yang et al., 2016; Papayannopoulos, 2018). In this work, we quantified the neutrophils’ viability using a trypan blue exclusion assay, and although there are other quantification methodologies considered more reliable and sensitive (Hosseinzadeh et al, 2012; Boros-Majewska et al., 2015), the robustness and consistency of the results justifies its validity. This observation reinforces the idea that yeasts induce a non-lytic NETosis, at least in the cases of *C. albicans* and *C. glabrata*. In what regard yeasts’ viability, a virulence-dependent profile in CFU counts was found with *S. cerevisiae* < *C. glabrata* < *C. albicans*. *C. albicans* showed the highest amount of non-adhered, -phagocytized, -contained yeast at 1 h p.i., and at 3 h p.i., the higher number of viable yeasts recovered after neutrophils uptake, emphasizing once more its virulent traits and ability to escape neutrophils immune response. Finally, although *Leishmania* nuclease expression (Guimarães-Costa et al., 2014) or *C. albicans* and Group A *Streptococcus* NETs degradation through DNase secretion were already described (Buchanan et al., 2006; Zhang et al., 2017), our results are also showing, for the first time, *C. albicans*, *C. glabrata*, and *S. cerevisiae’s* ability to degrade/digest DNA and NETs, *via* 3′NT/NU activity. As visualized in he gel runs and its quantifications, all yeasts were able to cleave NET-enriched supernatants, and this feature was prevented in the presence of TTM, a 3′NT/NU inhibitor. Also, the incubation with TTM of neutrophils infected with *C. albicans* resulted in a decrease of yeasts viability (p<0.05), turning *C. albicans* more prone to neutrophils control and elimination. Under these physiologic conditions, a pH of 7 to 8 was measured in NET-enriched supernatants, resembling pH values found in human blood. Although alkaline pH was associated to lower 3′NT/NU enzymatic activities in all yeasts tested, the complexity of these interactions seems to overcome this feature. However, we might speculate that, under acidic conditions, where the 3′NT/NU activity profile could be at its maximum, the yeasts’ enzymatic activity could be more pronounced. Moreover, whenever heat-killed yeasts were used, no degradation of NETs samples were obtained, and upon interaction with neutrophils, instead of NET formation, a more pronounced phagocytic profile was found. This indicates that neutrophils’ NETs extrusion upon contact with yeasts is dependent on *Candida* metabolism and not only on its recognition (Byrd et al., 2013; Hopke et al., 2016). This suggests the 3′NT activity as the mechanism responsible for these yeasts skills to digest NETs and highlights this pathway as a yeast cells’ strategy to escape to NETs killing.

The neutrophils’ innate immunity is pivotal in the resistance against opportunistic *C. albicans* and *C. glabrata* fungal pathogens, and the biological significance of NETs in fungal infections is thus just starting to be unfold. Here, *Candida* yeasts’ ability to hydrolyze and escape NET-mediated trapping and killing, through its 3′NT/NU enzymatic activity, was assessed and confirmed, raising however new questions to fully understand and characterize this NETs escape mechanism, namely if is entirely non-lytic and the molecular biology of this mechanism.

## Data Availability Statement

The original contributions presented in the study are included in the article/**Supplementary Material**. Further inquiries can be directed to the corresponding author.

## Ethics Statement

The studies involving human participants were reviewed and approved by the ethics committee of the FMUC. The patients/participants provided their written informed consent to participate in this study.

## Author Contributions

TG and LR designed the study. MA, AM, GS, AA, and LR performed the experiments. MA, TG, and LR analyzed the data and wrote the paper. RC, JM-F, TG, and LR edited and reviewed the final manuscript, with accordance of all authors. All authors contributed to the article and approved the submitted version.

## Funding

This work was financed by the European Regional Development Fund (ERDF), through the Centro 2020 Regional Operational Programme under project CENTRO-01-0246-FEDER-000010, CENTRO-01-0145-FEDER-000012-HealthyAging2020, and through the COMPETE 2020 - Operational Programme for Competitiveness and Internationalization and Portuguese national funds *via* FCT-Fundação para a Ciência e a Tecnologia, under projects POCI-01-0145-FEDER-007440 and UID/NEU/04539/2019.

## Conflict of Interest

The authors declare that the research was conducted in the absence of any commercial or financial relationships that could be construed as a potential conflict of interest.
