# Supplementary material for: Candida Extracellular Nucleotide Metabolism Promotes Neutrophils Extracellular Traps Escape
Source: Front Cell Infect Microbiol. 2021 Jul 13;11:678568. doi: 10.3389/fcimb.2021.678568 (PMC8313894; doi:10.3389/fcimb.2021.678568)
Supplement: Supplementary file 1 [file Table_1.docx]

Supplementary Material

# Supplementary Figures


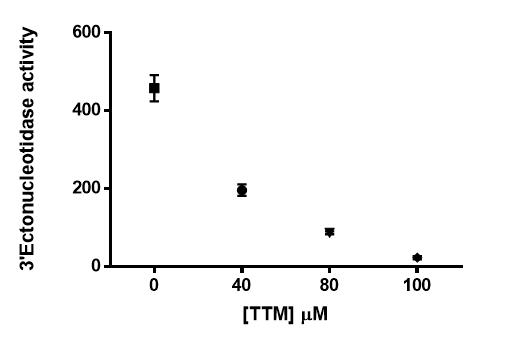


**Supplementary Figure 1.** ***Candida albicans* 3’-nucleotidase activity upon incubation with TTM.** Intact cells of *C. albicans* were allowed to cleave 3’AMP for 1h at room temperature, at different TTM concentrations. Data are shown as means ± SEM of at least three independent experiment.


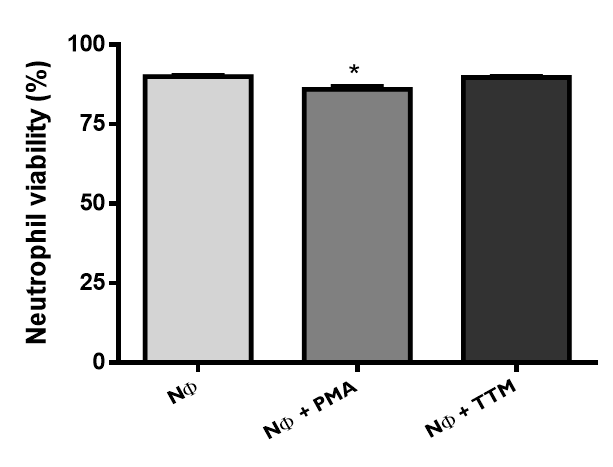


**Supplementary Figure 2.** **Percentage of neutrophils viability upon different conditions.** Trypan blue assays were performed, to distinguish between viable and non-viable neutrophils (Nɸ) at 1h and stimulated with PMA and TTM. Data represents at least three independent experiments (means ± SEM). *p<0.05.
